# SARS-CoV-2 Epidemics in Retirement and Nursing Homes in Italy: A New Preparedness Assessment Model after the First Epidemic Wave

**DOI:** 10.3390/ijerph18115712

**Published:** 2021-05-26

**Authors:** Carmelo Gugliotta, Davide Gentili, Silvia Marras, Marco Dettori, Pietro Paolo Muglia, Maria Giuseppina Desole, Marcello Acciaro, Sabina Bellu, Antonio Azara, Paolo Castiglia

**Affiliations:** 1 Department of Medical, Surgical and Experimental Sciences, University of Sassari, 07100 Sassari, Italy; d.gentili@studenti.uniss.it (D.G.); ma.silvia.87@gmail.com (S.M.); p.p.muglia@live.it (P.P.M.); azara@uniss.it (A.A.); castigli@uniss.it (P.C.); 2 ASSL Sassari, 07100 Sassari, Italy; mariagiuseppina.desole@atssardegna.it; 3 Regional Agency Emergency and Urgent Care Sardinia, AREUS, 07100 Sassari, Italy; marcello.acciaro@areus.sardegna.it; 4 University Hospital of Sassari, 07100 Sassari, Italy; sabina.bellu@aousassari.it

**Keywords:** COVID-19, preparedness, risk communication, nursing home, infection prevention and control

## Abstract

The aim of the study is to evaluate the preparedness of retirement and nursing homes in the city of Sassari at the end of the first wave of the severe acute respiratory syndrome coronavirus 2 epidemic, first by investigating the risk perception of epidemic outbreaks by the facility managers and subsequently by carrying out a field assessment of these facilities. To perform the field assessment, a checklist developed by the CDC (Infection Prevention and Control Assessment Tool for Nursing Homes Preparing for COVID-19) and adapted to the Italian context was used. Fourteen facilities took part in the survey (87.5%). The application of good practices for each survey area was expressed as a percentage with the following median values: restriction policies (87.5%), staff training (53.8%), resident training (67.6%), availability of personal protective equipment (41.7%), infection control practices (73.5%) and communication (80%). Among the facilities, considerable variability was observed in these evaluation fields: only the restriction policies and communication activities were applied uniformly. A discrepancy was found between perceived risk and real danger in the facilities, requiring targeted communication actions. At present, it is necessary to promote a new approach based on the prediction of critical events, thereby providing the means to effectively address them.

## 1. Introduction

Italy was the first European country to be severely affected by the coronavirus disease 19 (COVID-19) pandemic. According to data from the European Centre for Disease Prevention and Control (ECDC), Italian National Institute of Health (ISS) and the Italian National Institute of Statistics (ISTAT), by 20 March, Italy had reached the peak of COVID-19 cases during the first phase of the pandemic, surpassing that of China [1,2,3].

Pandemics affect a large proportion of the population and require a multisectoral response spanning months or even years. Preparedness for such an event is a continuous process of planning, exercising, revising and translating into action national and subnational response plans [4].

According to the United Nations Office for Humanitarian Affairs and the World Health Organization, the term ‘preparedness’ refers to the ability of governments, professional response organizations, communities and individuals to anticipate and respond effectively to the impact of likely, imminent or current hazards, events or conditions. An adequate level of preparedness can make a major difference in saving lives and reducing suffering. Moreover, it ensures that scarce resources are directed where they will have the greatest impact [5,6].

Being properly prepared for a major epidemic and pandemic event requires a combination of political will, financial investment, and public health expertise. To further improve the prevention, identification, and response to infectious disease outbreaks, it is essential to identify the most important challenges to address based on existing knowledge and previous experience of epidemic emergencies [7,8].

In the last two decades, the world has witnessed numerous major epidemics or pandemics, five of which (2009 H1N1 pandemic, 2014 Ebola virus epidemic, 2014 poliovirus epidemic, 2016 Zika virus epidemic, 2018 Ebola virus epidemic) have been declared “Health Emergencies of International Interest” by the World Health Organization (WHO). These events should remind us to remain extremely vigilant, taking advantage of interpandemic periods to improve preparedness and gain knowledge from past experiences. Moreover, a better preparedness should be sought as a priority, especially for the protection of vulnerable people [9].

During the emergency, numerous residential facilities for the elderly in Italy have been the *loci* of major outbreaks [10,11]. Furthermore, these structures also had a lower response capacity than hospital facilities to control the disease. The biggest issues were the lower provision of personal protective equipment, the lack of specific preparedness, and a logistical–structural organization that often failed to set up adequate spaces to isolate sick residents [12,13].

Data from a survey by the Italian National Institute of Health (ISS) shows that in the first half of 2020, in Italy, more than 7% of the total number of deaths in residential facilities for the elderly involved residents infected with SARS-CoV-2 and that over 30% of the death toll had COVID-like symptoms [10]. During the same period, Sardinia showed a low risk for COVID-19 cases and COVID-19 mortality [14] but the number of cases among residents in long-term care facilities accounted for more than 40% of the total registered COVID-19 cases [15].

In addition to advanced age, residents in these long-term care facilities have a further risk of encountering unfavorable outcomes, due to the presence of comorbidities and chronic diseases. Moreover, these conditions often make it difficult to recognize the typical symptoms of COVID-19 [16,17].

For these reasons, retirement and nursing homes are a reference setting for public health initiatives aimed at supporting the best practices of infection prevention and control and reducing the likelihood of outbreaks in these facilities.

A guiding principle for any public health initiative in the first place is to set up an adequate communication strategy. During a risk communication process, the first task is to ascertain the real risk within a target population. The hazards are not always commensurate to the actual risks: outrage, an emotional factor that influences risk perception, affects people’s perception of the real danger of an event [18,19,20,21,22,23,24].

In the different risk communication scenarios, a targeted approach is required, and effective risk communication is essential to limit morbidity and mortality caused by communicable diseases, in addition to enabling the appropriate management of available resources [25,26]. The ability to provide timely and effective responses at an early stage, despite the scientific uncertainties and different resource capacities, is a key element to dealing with a public health crisis. Preparedness assessment should be carried out at all levels of a healthcare organization, including in residential facilities responsible for assisting fragile people such as nursing homes and retirement homes.

Finally, standardized tools for assessing preparedness in residential facilities for the elderly at national and subnational levels are lacking, and infection prevention and control practices are not routinely monitored.

Based on these premises, the aim of this study is primarily to evaluate perceived risk by all residential facilities for the elderly in the city of Sassari following the first wave of the SARS-Cov-2 epidemic, and subsequently, through a field inspection of each facility, to identify critical control points in order to avoid new outbreaks and provide an intervention model that could be used for a large-scale preparedness assessment of these facilities.

## 2. Materials and Methods

The present study did not require ethical approval for its observational design according to Italian law (Gazzetta Ufficiale no. 76 dated 31 March 2008).

### 2.1. Study Setting

Sardinia was one of the most affected areas of the country during the first epidemic wave considering its low population density. Even though age-adjusted attack rates in the region were classified as low [27] and the COVID-19 standardized mortality rate was lower than the Italian one [14], there was an overall increase in mortality of 13.7% in March 2020 compared with the average value in the same reference period for the years 2015 to 2019 [28]. Moreover, due to its insularity, Sardinia has particular genetic features that make its population suitable for epidemiological studies [29,30,31].

The island is located in the Mediterranean Sea, over an area of 24,100 Km^2^. The region is divided into four provinces (Nuoro, Oristano, Sassari, Sud Sardegna) and a metropolitan city (Metropolitan City of Cagliari). Sardinia has the oldest population in Italy: according to data provided by the National Statistical Institute, as of 1 January 2020 (Table 1), the average age of residents was 47.3 years (the Italian average age was 45.7 years). The province of Sassari has the lowest old-age index (202.7) in the region, but this value is higher than the index detected at the national level (178.4) [32].

The first case of SARS-CoV-2 infection in the island was reported on 2 March in the metropolitan area of Cagliari. In the following days, there was a moderate increase in the number of cases. On 14 March, the first hospital outbreak occurred in the cardiology ward of the University Hospital of Sassari. As of that date, 77 cases had been identified on the island, 35 of which were reported in the province of Sassari, making it the most affected area in the region [33,34].

**Figure 1:**
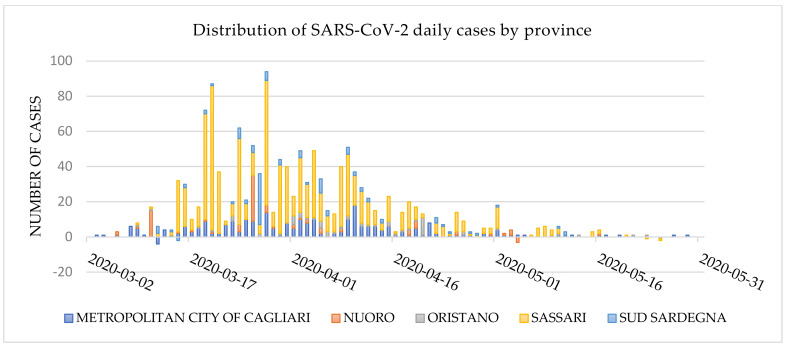
Daily distribution of SARS-CoV-2 cases by Sardinian district as of 31 May 2020 (data source: Civil Protection Database).

The district of Sassari was the most affected area throughout the first wave of the COVID-19 epidemic. As of 31 May 2020, 1356 cases were identified in the region, 64.2% (870) of which were in Sassari (Figure 1), and more than 40% among residents in long-term care facilities [15].

### 2.2. Questionnaire Investigation

In June 2020, a survey was carried out in the city of Sassari to identify all the residential facilities that provided assistance to the elderly and infirm in the city. The investigation identified 16 residential facilities, two of which were nursing homes. Information was gathered regarding the epidemiological situation inside these facilities and the spread of SARS-CoV-2 in the March to May 2020 timeframe.

A letter describing the objectives of the project, the stakeholders involved, and the survey methods was sent to these facilities. Commitment to the project was requested through the completion of a digital survey by the head of the facility (Supplementary Materials, Table S1), using the EUSurvey platform (the European Commission’s official survey management tool). To refine and validate the survey, a two-stage empirical validation study was conducted. This consisted of a pretest with a panel of domain experts, followed by a discussion on the questionnaire with the manager and the healthcare staff of a facility for the elderly not included in our project.

The purpose of this preliminary assessment was mainly exploratory and involved gathering information about the organization and the management of the facilities during the first phase of the COVID-19 emergency. This step also marked the first contact between the group responsible for the investigation and the residential structures involved in the project.

### 2.3. ICAR Tool

The Infection Control Assessment and Response Tool (ICAR), developed by the US Center for Disease Control and Prevention, was used for the field investigation to assess a healthcare facility’s infection prevention and control (IPC) practices and to guide quality improvement activities (by identifying and addressing gaps) in a standardized manner [35].

The ICAR checklist was adapted to the Italian context according to regulations provided by the Ministry of Health and the National Institute of Health (ISS) on the management of residential facilities for the elderly [36]. In particular, facility access restrictions were brought into line with the national law, cloth face coverings were not permitted in place of facemasks, the minimum distance between residents was set at 1 m (wherever possible, 1.8 m) and facilities’ chains of communication for suspected or confirmed cases of COVID-19 were adjusted to the regional health organization (family doctors of the residents, as well as health department, must be promptly informed).

The items assessed by the ICAR checklist included key strategies for keeping COVID-19 out of the facility, identifying infections as early as possible, preventing the spread of infections within the facility, assessing, and optimizing personal protective equipment (PPE) supplies and identifying and managing severe illness in residents with COVID-19. These items are grouped into six areas for monitoring: restrictions on visitors and non-essential personnel; education, monitoring, and screening of healthcare personnel (HCP); education, monitoring, screening and cohorting of residents; availability of PPE and other supplies; infection prevention and control (IPC) practices; and communication. A detailed explanation of each assessment area is featured on the original CDC checklist [35].

### 2.4. Field Investigation

The field investigation provided an opportunity for dialogue and information sharing, emphasizing that it was not a regulatory inspection and was designed to ensure the facility was prepared to quickly identify and prevent the spread of COVID-19. A team of four medical doctors, residents in Hygiene and Preventive Medicine from the University of Sassari and a nurse specialized in infection control risk assessment were set up for the task. The team underwent a week of training, with the aim of minimizing any variability during the field investigation. Every item of the checklist was tested, identifying shared methods of detection for each element of the ICAR. Each member of the investigation team was then assigned a specific section of the ICAR checklist to study and discuss.

The field investigation was carried out from mid-June to the end of July 2020 with a single visit for each facility and after having received a reply to the introductory questionnaire. Interviews were carried out with the facilities’ managers and COVID-19 representatives, staff on duty at the time of the survey and with the staff responsible for cleaning. In each facility, the visit was carried out with a direct observation of the assistance areas, the isolation/quarantine rooms, and residents’ shared areas, including the dining area and the spaces reserved for staff. Routine activities of health-care workers were also observed, especially those concerning infection prevention and control practices (IPC). In addition to the information collected, the facility manager and/or the person in charge of COVID-19 procedures were asked if a written plan had been drawn up specifically for the Sars-Cov-2 emergency and if the competent health authorities had transmitted information on the epidemic prior to the local emergency.

### 2.5. Statistical Analysis

At the end of each field investigation, the completeness of the information collected and the consistency of data between each team member were verified in a briefing. Subsequently, data were entered into a Microsoft Excel worksheet by two members of the team and analyzed using the IBM SPSS Version 20 statistical software. The Mann–Whitney U test was used to compare the distributions of the ICAR checklist scores between facility groups with and without previous SARS-CoV-2 cases.

For every facility in each macro-area of the ICAR checklist, a score was calculated from the ratio between the sum of the individual items in that area (a value of 1 point was attributed if the element was present) and the number of items in the section, reported as a percentage. In the exact same way, a global score for each facility was obtained, taking into consideration all six areas of the ICAR checklist, also reported as a percentage.

In addition, an evaluation of risk perception versus actual hazard in the facilities was made so as to formulate subsequent risk communication strategies. One point was given for each item fulfilled in IPC practices, quarantine/isolation and training areas (Table 2), both in the introductory questionnaire and the ICAR checklist used for the field assessment.

The perception of safety by each facility was considered as a proxy for assessing the perceived risk (perceived safety = 1 perceived risk). Likewise, the score of the external evaluation depicts the complementary value to the real danger assessed. Our graphical representation of these variables therefore shows an inversion in the XY axis compared to the classic outrage–hazard Sandman model.

Three pairs of indicators were devised to compare the risk perceived by the facilities (evaluated by items extracted from the introductory questionnaire) with the danger assessed by the team during the field investigation (evaluated by items extracted from the ICAR checklist) in each area. Each indicator is the percentage of items fulfilled for each of the three areas of the questionnaire and the ICAR checklist.

## 3. Results

### 3.1. Introductory Questionnaire Results

Fourteen of the 16 facilities (87.5%) identified within the city of Sassari participated in the project. Two of these are nursing homes (14.3%), while the remaining are retirement homes (85.7%). The sample comprises structures with heterogeneous structural characteristics. There are both small (a limited number of beds and residential rooms) and large structures. The total number of beds available in the 14 facilities is 790 units (median 40; range of values 15 to 136).

At the beginning of the survey, 88.8% of the beds were occupied by 617 residents. Out of the total of 591 rooms in these facilities (median 24.5; range 12 to 117), 41.6% (246) are single rooms (median 9; range 0 to 64). According to data reported by the facilities, 45 beds had been earmarked for quarantining new inhabitants (5.7% of the total number of beds) and 39 for isolating possible, probable, or confirmed cases (4.9%). At the time of data collection, there were 47 nurses (9.4%) and 224 social and health workers (44.7%) out of the total employees in the 14 facilities.

Prior to this study, two facilities had had a SARS-CoV-2 outbreak with a total of 251 cases among residents and healthcare workers. The period prevalence of infection in the two facilities was 58.8% (164 positives out of 279) among residents and 30.2% among the healthcare workers (87 positives out of 288), with a significant difference between the groups (*p* < 0.05). Demographic information, lethality by age and gender, and comorbidity of SARS-CoV-2 cases are reported in Table 3.

The mean number of comorbidities found is 2.5 ± 1.6. Similar data has already been reported in previous experiences, aimed at examining the health conditions of subjects who had died of COVID-19 [15]. By evaluating the comorbidity classified as “other conditions”, a misclassification was detected which could weigh on an underestimation of the other classes of comorbidity (especially for chronic neurological diseases).

At the time of the survey, no facility had any cases of SARS-CoV-2 infection among residents and staff.

The facilities were able to report adherence to infection prevention and control practices together with procedures that had been implemented since the beginning of the state of emergency by the pre-audit questionnaire. For some of these practices, the facilities reported 100% adhesion (ABHR dispenser available at the entrance of the facility, instructing facility staff to stay at home if sick, restricting visitation in the facility, use of surgical masks by visitors, screening of staff at the entrance of the facility by measuring body temperature, facility staff informed on the procedures for admitting a new resident; facility staff informed about social distancing, facility staff informed on procedures to adopt for a possible or probable COVID-19 case, periodic sanitation of workers’ premises, presence of a supervisor for monitoring consumption and purchase of PPE; ABHR available to all facility staff, rapid isolation procedures and reporting of possible, probable or confirmed cases).

The lowest adhesions were reported in the provision of an area for cohort isolation of patients and for putting in place a procedure for transferring cases whenever effective isolation could not be provided (64.3% of the facilities). Specific training for COVID-19 was reported in 10 facilities (75.4%) with involvement of more than 75% of the staff in only eight of these structures (Table 4).

### 3.2. Field Assessment Results

The 66 items assessed by the ICAR checklist were divided into six specific macro-areas (Table 5).

#### 3.2.1. Visit Restrictions and Non-Essential Personnel Restrictions

All facilities (100%) had limited visitors’ access; they had suspended non-essential services and simultaneously activated alternative methods to visiting (operator-assisted video call was guaranteed in all facilities). In half of the residences, under special circumstances, visitors could access the facilities for exceptional assistance requirements (compassionate assistance and end of life). These exceptions, where provided, could only be approved by the manager of the structure. Communications regarding the new visitation policies of the facilities were sent by 12 residences (86%) to all the residents’ families by e-mail or by telephone. Twelve residences were equipped to screen visitors by measuring body temperature at the entrance of the facility and provided behavioral constraints during the visit (movement inside the facility limited to the resident’s room or to a designated location, hand hygiene at the entrance and the obligation to wear a surgical mask for the entire duration of the visit). Only at the entrances of three structures (21%) were there notices prohibiting access to visitors.

#### 3.2.2. Education, Monitoring, and Screening of Healthcare Personnel (HCP)

This area aims to assess the activities that reduce the likelihood of healthcare staff acting as disease vectors within the facility. Only six structures (43%) provided specific training on COVID-19, while information regarding new infection control policies was given in 71% of the structures and information about sick leave was provided for every facility. Internal audit activities concerning the adherence of facility staff to hand hygiene, the correct use of PPE and cleaning practices were carried out in 14% of the facilities. Moreover, the assessment of staff availability and an established recruitment method in the event of under-staffing was carried out only in five structures (36%). The universal use of face masks by the facility staff was implemented in 12 residences for the elderly, but adequate information on the different types of face masks to be used during care assistance was given only by six facilities. Nearly 80% of the facilities screen staff by checking body temperature and COVID-19 symptoms at the beginning of the work shift. With the same frequency, social distancing between the facility staff is reiterated unless necessary for patient care.

There seems to be a clear direction to remove from the workplace staff who manifest symptoms of COVID-19 during daily activities or at the time of screening for body temperature and symptoms (86%) while in a smaller number of facilities (43%) any staff found to be symptomatic were documented.

#### 3.2.3. Education, Monitoring, and Screening, and Cohorting of Residents

As in the previous section, the practices that reduce the likelihood of residents acting as infection vectors were identified. All the facilities made efforts to educate the residents to some extent about the disease, the symptoms, the actions that guests could take to protect themselves from infection and the actions that the staff was taking to protect them. However, the transmission of information to residents appears to be limited by the ability of the residents to manage the concepts provided by the facility staff.

Only 10 out of 14 structures had started a daily screening of residents for body temperature but almost all were aware of the need to adopt appropriate precautions to the residents even in the occasional finding of symptoms. In 10 facilities (71%), there was a register of symptomatic residents or an annotation was made in the clinical diary. Internal limitations on the movement of residents were poorly applied and shared catering was active in most of the facilities, also due to the absence of active cases at the time of the survey.

Ten structures (71%) had a dedicated area of the building for COVID-19 cases. Only two facilities (14%) had planned to dedicate specific personnel to COVID-19 areas. A plan for the management of patients who develop COVID-19 was available in nine facilities while only eight structures had provided a plan for incoming residents with an unknown COVID-19 status.

#### 3.2.4. Availability of Personal Protective (PPE) Equipment and other Supplies

The main objective was to verify that an assessment had been carried out on the stocks of PPE available and that these stocks were sufficient to deal with a shortage situation on the market, as had occurred in an initial phase of the emergency. Adequate assessment had been performed in only 10 facilities (71%). For any shortage of PPE, the involvement of the Prevention Department or the Health District was considered only in 36% of cases.

Half of the facilities had adopted procedures to optimize the use of PPE (priority access to health care personnel, based on patient care procedures, extended use of the devices). None of the investigated structures had an available kit of personal protective equipment near the assistance area or near the residents’ rooms (it was mainly available in the staff changing areas or given to the staff by the person in charge for PPE management where present). All the facilities had acquired hospital-grade disinfectants with virucidal action on SARS-CoV-2 for the disinfection of surfaces, environments, and equipment. Only 36% of the facilities had made disposable wipes and easily accessible bins available in the common areas, promoting good respiratory hygiene practices.

#### 3.2.5. Infection Prevention and Control Practices

This section of the checklist, relating to infection prevention and control (IPC) practices, includes items that investigate the knowledge of staff on hand hygiene with particular focus on the five hand hygiene moments identified by the World Health Organization, on the use of PPE for possible, probable, or confirmed COVID-19 cases and regarding the appropriate use of disinfectants. The moments with greater awareness of the usefulness of hand hygiene are before contact, after body fluid exposure risk and after PPE removal. Less attention is paid after contact with the patient and before a clean/aseptic maneuver. Only four facilities reported a preferred use of ABHR compared to washing hands with soap and water, although half of the facilities had made supplies of ABHR available and easily accessible in the care areas. Almost all the facilities’ staff were aware of which PPE to use in assisting a possible, probable, or confirmed case of COVID-19, with greater concern given to the use of gloves (in 100% of the facilities, it was reported as fundamental PPE). Monitoring of compliance in the practice of hand hygiene and regarding the correct use of PPE was carried out in only two facilities (14%). As far as the disinfection of the rooms is concerned, all the residences use suitable disinfectants against SARS-CoV-2 but only four facilities (29%) were aware of the need to apply the cleaning product for an adequate contact time.

#### 3.2.6. Communication

The survey showed a good communication capacity of the structures during the emergency. The facilities demonstrate that they had followed the instructions provided by the local health authorities for reporting possible, probable, or confirmed cases of COVID-19, including serious respiratory infections that had resulted in hospitalization or death of the resident. Clusters of new onset respiratory symptoms among residents or health personnel (two or more cases in 48 h) were frequently reported by the facilities (86%).

In 93% of the sample analyzed, there was a procedure to inform residents, families, and facility staff members about suspected or confirmed cases of COVID-19. Finally, in nine out of 14 structures (64%), the communication of a possible, probable, or confirmed COVID-19 case was envisaged before the transfer of a resident to a hospital, even for chronic treatments such as dialysis (Table 5).

A specific written plan for the COVID-19 emergency was identified in only four structures of the 14 interviewed. All the facilities reported a lack of information at the beginning of the health emergency with only five facility managers reporting having received information before the epidemic period (only in three cases had such information been received from the local health authority).

**Figure 2:**
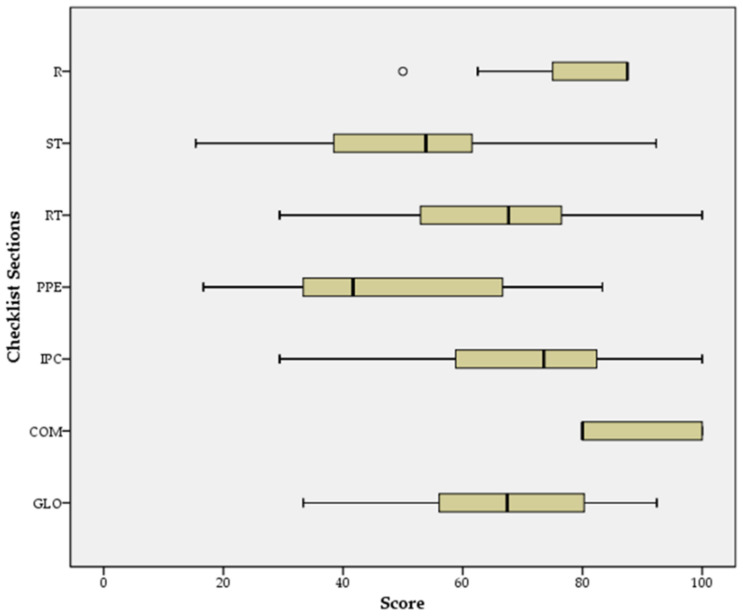
Distribution of facility scores by checklist section (R: restrictions policies for visitors and non-essential staff; ST: staff training, monitoring, and screening; RT: resident training, monitoring, and screening; PPE: Availability of PPE and other supplies; IPC: infection prevention and control (IPC) practices; COM: communication; GLO: global checklist score).

The analysis of the scores obtained by the facilities in every section of the checklist highlights a great variability between the scores for staff training, monitoring and screening; resident training, monitoring and screening; availability of PPE and other supplies; infection prevention and control practices. Conversely, visitor restrictions and communication areas show low variability in scores (Figure 2).

**Figure 3:**
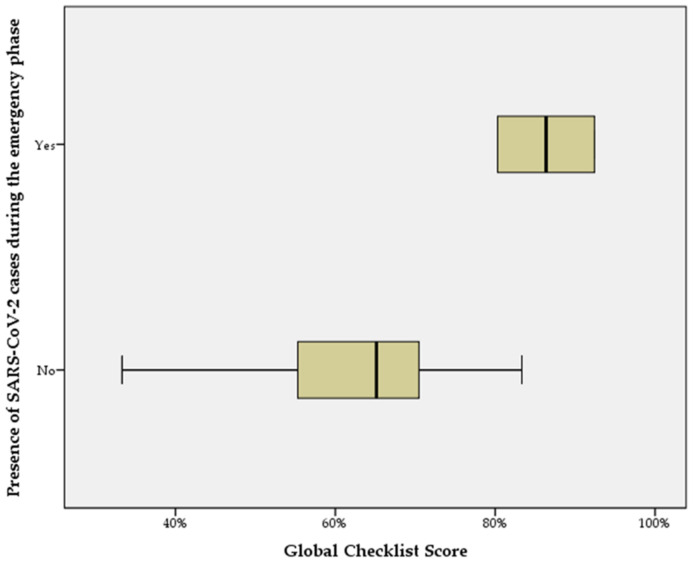
Distribution of total checklist scores obtained by facilities according to the presence of SARS-CoV-2 cases during the emergency phase.

When the global checklist score distributions are compared according to the independent categorical variable “presence of SARS-CoV-2 cases during the emergency phase”, the Mann–Whitney U test shows a statistically significant difference between the facility groups (*p* < 0.05) (Figure 3).

### 3.3. Risk Perception Versus Facilities Hazard Analysis

The three pairs of indicators that were built to compare risk perception versus actual hazards regarding IPC practices, quarantine/isolation and training areas, highlighted a potential incongruity between risk perception and actual hazard by the facilities.

**Figure 4:**
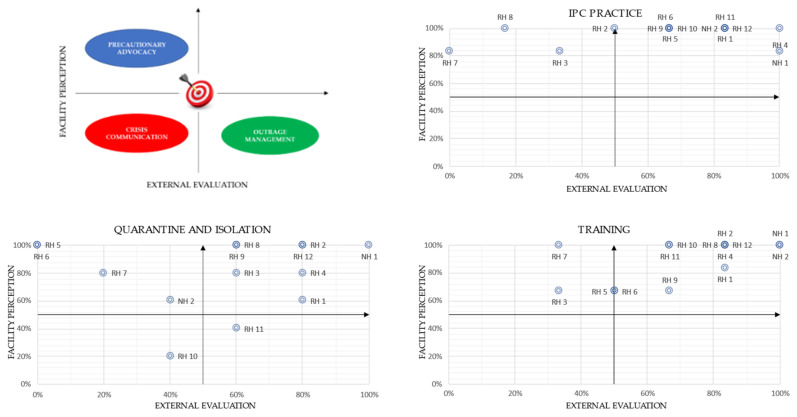
Perceptual map of risk (facility perception) versus hazard (external evaluation) for the IPC practice indicator, quarantine/isolation indicator and training indicator.

A high perception (low outrage) was found for four facilities in the application of IPC practices (Figure 4, IPC practice plot, upper-left-side quadrant). However, this perception was not consistent with the subsequent field investigation, where an adequate application of IPC practices was not found. For the other facilities, perception was consistent with external evaluation.

As regards quarantine/isolation indicators (Figure 4, quarantine and isolation plot), four facilities (upper-left-side quadrant) show a low risk perception not commensurate to the real hazard assessed by the external evaluation. One facility (RH10) showed a high outrage in these practices, which was proportionate with the level of danger assessed during the field investigation. Lastly, one facility (RH11), despite having an adequate isolation and quarantine competence, showed a high outrage on these items without having a high hazard.

In the area concerning healthcare staff training (Figure 4, training plot), the field investigation encountered a lack of training in at least two facilities (RH3-RH7), with extensive room for improvement by all retirement homes. On the other hand, most of the facilities reveal a high perception of the training activity carried out. Nursing homes showed a higher level of training and a commensurate perception of the same.

## 4. Discussion

Planning is a fundamental component of preparedness but does not consist solely of drawing up written plans. It is now widely recognized that preparedness must include a vulnerability assessment, skills evaluation, and training programs, and must strengthen the communication network. All these activities must be implemented in the planning phase. An adequate level of preparedness can be achieved only by providing all the tools necessary to develop an adequate response to the critical event [37].

In March 2020 in the city of Sassari, the Local Public Health Service of the Prevention Department issued a questionnaire to investigate the supply of personal protective equipment within the residential facilities. Nine of these facilities replied, reporting shortages of face masks (55.6%), gowns (44.4%), gloves, environmental sanitation products and products for disinfection and hand hygiene (22.2%).

Beyond this snapshot assessment of available personal protective equipment and disinfectants, residential facilities were not routinely evaluated in infection prevention and control practices and in emergency response capacity, mainly because a dedicated monitoring system for these facilities is lacking.

An adequate level of staff and resident education, regular monitoring of care standards, improvements in hand and respiratory hygiene, the provision of adequate stocks of PPE devices and the promotion of social distancing are strong drivers to avoid an outbreak in a facility. Such measures should be supported by the ability to detect suspected cases of infection early and isolate them promptly and properly [38,39]. In our experience, it has been shown that the application of some measures was incomplete. Facilities complied to a greater extent with measures such as restrictions for visitors and timely communication of possible, probable, or confirmed COVID-19 cases. An explanation for such a high level of adherence is partly due to national regulations that imposed restrictions on visits and clear information on the need to immediately notify the residents’ family doctor and the local health authorities of suspected or confirmed cases of infection [40,41].

The preventive lockdown of facilities, although effective, must be complemented by a set of other measures that limit the probability of SARS-CoV-2 infections within the structures. This probability is reduced as adherence to infection prevention and control practices increases. Nonetheless, there is no single procedure that can single-handedly lower the risk threshold to zero. A common finding was the failure to set up an adequate screening procedure before allowing access to the facilities, both for staff and, occasionally, for authorized visitors. This is partly due to a low perception of the real danger brought about by the sense of security given by the restrictions on visits from outsiders. When an entry screening is carried out, it is essentially based on measuring body temperature, but this assessment would be more effective if associated with an anamnestic interview seeking epidemiological information as well as other symptoms that can occur (e.g., sore throat, runny nose, anosmia, dysgeusia) [42].

Equally important is the daily symptom screening in residents, although this is made difficult by the frequent presence of other comorbidities, which makes early recognition of respiratory viral infections difficult, potentially delaying diagnosis and increasing the probability of viral spread [43]. Although a periodic testing strategy could provide greater guarantees (diagnosis of asymptomatic SARS-CoV-2 cases), daily symptom screening should not be neglected.

A risk analysis and management model that could effectively explain accident causation is the one predicted in Reason’s Swiss cheese model, which has been applied several times to the hospital setting and that can be applied in our intervention setting [44]. The underlying assumptions of this model provide a system approach: a fallacy is more a consequence than the cause of the problem. Lapses and weaknesses in one defense do not allow a risk to materialize, since the presence of a single weak spot usually does not alone cause an outbreak, but if all the barriers have leak points (latent or active conditions), the ideal condition for the perfect storm may occur [45].

The prediction of latent conditions is therefore the foundation for ensuring a proper responsiveness of the system. For example, eight facilities have a specific procedure on new hospitalizations but just a few have provided for a quarantine period for the new resident, relying on the negative result of the diagnostic molecular test without ensuring a period of social distancing from the other inhabitants. Regardless of which of the two procedures is the most effective in reducing the transmission of infection, the perception of the effectiveness of the quarantine period and the diagnostic test usage are different within facilities without scientific evidence or on an assessment of the risk [46].

Moreover, a lack of specific training gives rise to an incongruity between perceived risk and actual hazard. This gap is also exacerbated by the failure of the facilities to provide written plans stating their practices and supported by scientific evidence. A specific plan, besides being essential for standardizing the daily practice of personnel working in the facility, is also a useful tool for educating new staff on the procedures to be adopted. The plan is not to be considered as a dogma, but rather, needs to be updated as new evidence is acquired. The lived experience of an epidemic outbreak seems to have significantly influenced the application of the procedures. Previous experience in dealing with a catastrophic event improves the ability of an organization to react to a new event [47]. This suggests that the public health authorities, who have the correct knowledge of the risk, should cooperate with the structures facing emergencies by increasing the capacity of the individual structures and assessing their specific characteristics (e.g., structural, managerial, organizational) [48]. In this regard, logistical–structural characteristics of the building are a fundamental element to allow adequate spaces for isolation and quarantine.

A clear knowledge of the responsiveness of each facility is a fundamental preparatory step for an adequate risk communication process and regarding the ability to put in place all procedures aimed at reducing the risk itself.

Management and coordination of healthcare professionals is essential to ensure an effective and timely response during an outbreak. Nurses and healthcare assistants are the largest group of health professionals in these facilities, and it is therefore essential that their training provides a good basic knowledge of IPC and health emergency management. The assessment of basic skills in disaster medicine is identified as a means capable of ensuring the application of the best health care practices during an emergency or a health crisis [49]. The core competencies comprise a broad set of indicators that describe all the skills needed by healthcare professionals to be able to confront the broad discipline of disaster medicine [50]. In particular, interventions implemented at the onset of an epidemic such as health monitoring and surveillance, epidemiological investigation, isolation and quarantine, fast and wide testing, protection of first responders and health workers and basic sanitation and hygiene, storage, distribution and dispensing of supplies are critical to damage control. This also requires basic knowledge of descriptive and analytical epidemiology, laboratory science, environmental and occupational health, infection control, effective communication practices, and social sciences.

The professionals involved should be able to support surveillance efforts and be familiar with the arrangements and procedures for reporting cases. Learning the basics of risk and health communication is crucial to informing affected individuals, their families and the media regarding exposure risks and potential preventive measures.

Finally, health professionals need to know the moral, ethical, and legal issues that are relevant to the management of affected populations and communities and the basic public health regulatory framework. Well-defined ethical principles must underlie decision-making processes in emergency situations (for example, decisions on the allocation or use of insufficient resources) [51].

Regarding the knowledge related to SARS-CoV-2 and IPC practices, we found that only six facilities had carried out specific training and, in most cases, this was attended by the facility staff on a voluntary basis.

Moreover, IPC measures were applied without a real understanding of the reasons that led to their application. Training on the causative agent responsible for the epidemic is essential as it enables staff to acquire new knowledge and deepen existing knowledge about the transmission paths, clinical manifestations, incubation times, virulence, infectivity, contagiousness, sources and reservoirs of infection. Knowledge of these characteristics allows the application of effective preventive measures (for example, an adequate isolation or use of PPE) and to promptly recognize suspicious cases to be sent for testing.

Nursing homes, providing accommodation to people with high clinical complexity, were inclined to provide adequate training to employees.

The training took place via e-learning and the main web platform used was provided by the National Institute of Heath. The main reasons for using this web platform were free usage, easy accessibility and advice from third parties. However, healthcare assistants expressed difficulties in the availability of this e-learning since access was restricted to nurses and medical doctors. The activity in the field made it possible to supply some knowledge but it is essential to organize an extensive training program capable of overcoming the shortcomings and weaknesses of healthcare professionals working in these sectors.

The field investigation was an effective method for clarifying how much more can be carried out to improve risk communication and evidence-based procedure in these facilities. One approach could be legislative, with a revision of the regional laws identifying people who are responsible for assessing preparedness in nursing homes and retirement homes. Providing a committee for healthcare-related infections such as that in hospitals or the provision of a medical director in residential facilities (currently present only in nursing homes) could significantly improve the safety of these facilities.

Effective communication between all stakeholders is vital for decision-making. Risk communication can be defined as the exchange of information and risk assessments between experts, legislative authorities, interest groups and citizens [52]. The objectives of this type of communication can be manifold: it can be used to reassure people or stimulate risk perception, to guide choices or motivate the population to apply precautions. A precondition of both types of communication is credibility. The “Crisis and Emergency Risk Communication” (CERC) model developed by the US CDC integrates the two approaches described above in a single multiphase communication model to be used in the context of health emergencies [53].

The CERC model can help health organizations to provide the public with information to make the best decisions and to accept the imperfect nature of choice by implementing six principles of effective emergency and risk communication:

- Be first: crises are time sensitive. Communicating information quickly is crucial. For citizens, the first source of information often becomes the preferred source.
- Be right: accuracy establishes credibility. Information can include what is known, what is not known, and what is being done to fill in the gaps.
- Be credible: honesty and truthfulness should not be compromised during crises.
- Express empathy: crises create harm, and the suffering should be acknowledged in words. Addressing what people are feeling, and the challenges they face, builds trust and rapport.
- Promote action: giving people meaningful things to do calms anxiety, helps restore order, and promotes some sense of control.
- Show respect: respectful communication is particularly important when people feel vulnerable. Respectful communication promotes cooperation.

The risk is having to fight, even before the pandemic, with infodemics or the viral spread of false, partial, or erroneous information [54]. Immediate and widespread sharing of information offered by digital and social media before it has been thoroughly verified can be dangerous (for example, the non-evidence-based use of drugs such as hydroxychloroquine) [55,56].

This study involved a small sample of residential facilities for the elderly, although representative of one of the major cities in the Sardinian island, and a high response rate was achieved. A close deadline was given to complete the preliminary questionnaire and the field investigation was performed within a month, making it unlikely that facilities were influenced by the responses given by the other facilities.

The main limitation of this study consists in possible confounding factors due to the heterogeneous logistical–structural characteristics among the facilities, which may have played an important role in the genesis of SARS-CoV-2 outbreaks. It is also possible that the perceived risk observed was influenced by recent SARS-CoV-2 epidemics among the facilities, which may have expressed a perception of risk different from the usual one. Finally, this public health action would be difficult to manage on a large scale during an epidemic phase, especially due to the lack of human resources, indicating its use in a pre-epidemic period.

An unexpected element of our intervention was the willingness of the managers and facilities staff to open up to discussion on aspects not considered in the planning phase, in order to guarantee a better quality of care for their residents, and many expressed a readiness to collaborate in a subsequent training project.

The field investigation, using an interactive method of detection, made it possible to convey some information and advice regarding the management of infectious events to the facility staff and managers. The ICAR checklist made it possible to clearly identify the weaknesses in the facilities’ defensive systems and can be used to recommend practices to be implemented.

In light of what has been observed and discussed, it seems appropriate to draw attention to the fact that in every crisis, leaders have two equally important responsibilities: solving the immediate problem and preventing it from happening again [57].

## 5. Conclusions

This study highlights how important it is for health authorities to acquire knowledge about risk perception among a population during an epidemic event, and compare it with the real hazard, so as to design a more effective public health response.

The method used for this investigation of nursing homes and retirement homes allowed a reliable and fast evaluation of the procedures implemented by the facilities.

To our best knowledge, this is the first field investigation that aims to assess the risk perception of COVID-19 outbreaks among retirement and nursing homes, comparing it with actual danger.

The new epidemic wave forces us to focus our attention on the present, but it is necessary to develop current plans by projecting ourselves into the future and promoting a new approach based on the prediction of critical events, providing the means to effectively address them. Avoiding this challenge is no longer an option. The ongoing pandemic, with its second and eventually third wave, will make it possible to evaluate in the near future the effectiveness of the interventions initiated in retirement and nursing homes who have joined the project, both in terms of the number of facilities affected by the penetration of the infection and in terms of cases that will be registered within them.

## Figures and Tables

**Table 1:** Istat demographic indicators 2020.

	Italy	Sardinia	Sassari	Nuoro	Cagliari	Oristano	Sud Sardegna
Old-age index (%)	178.4	221.6	202.7	216.7	204.8	272.8	255.4
Average age of the population	45.7	47.3	46.6	47.1	46.7	48.8	48.3

**Table 2:** Indicators of risk perception versus facilities hazard assessment (each item was given one point if performed).

Facilities’ Perception	Assessment of Facilities
(Items Extracted from Introductory Questionnaire)	(Items Extracted from the ICAR Checklist)
IPC Practices	
Body temperature is monitored for all staff before entering the facility	All HCP (including everyone in the facility staff) are screened at the beginning of their shift for fever and symptoms of COVID-19
At the entrance of the facility, alcohol-based hand rub (ABHR) is available for everyone	Potential visitors are screened prior to entry for fever or symptoms of COVID-19. Those with symptoms are not permitted to enter the facility
Anyone entering the facility wears a surgical mask for the entire length of stay	Visitors that are permitted inside must wear a cloth face covering while in the building and restrict their visit to the resident’s room or other location designated by the facility. They are also reminded to frequently perform hand hygiene
Body temperature is measured daily for all the residents of the facility	The facility assesses residents for fever and symptoms of COVID-19 upon admission and at least daily throughout their stay in the facility
A facility staff member has been identified to verify the correct use of PPE by health workers and proper hand hygiene	The facility monitors HCP adherence to recommended IPC practices
The ABHR is readily available to all healthcare staff during patient care activities	PPE is available in resident care areas including outside resident rooms
Quarantine/Isolation	
Newly admitted residents are housed in a dedicated room and avoid contact with other people during the observation period (14 days)	The facility has a plan for managing new admissions and readmissions whose COVID-19 status is unknown
Newly admitted residents are swabbed before entering the facility and at the end of the observation period (14 days)	The facility has dedicated a team of primary HCP staff to work only in this area of the facility
There is a procedure for rapid isolation of people with a new confirmed/suspected case of COVID-19 and the case is reported to the referring physician	The facility has a plan for how to deal with residents in the facility who develop COVID-19
Transfer of a COVID-19 confirmed/suspected case to another facility is expected if adequate isolation of the subject cannot be arranged	The facility has dedicated a space in which to care for residents with confirmed COVID-19.
A dedicated area of the facility has been identified and could be used in case of a high number of COVID-19 cases (cohort isolation)	Availability of beds in isolation facilities assessed at time of survey
Training	
Facility staff (including non-employees) are expressly informed not to attend work with fever, myalgia, asthenia or respiratory/gastrointestinal symptoms	The facility has provided education and refresher training to HCP (including consultant personnel) about the following: COVID-19
The facility staff has been informed to avoid close contact between colleagues, except for any resident care activity if necessary	The facility has provided education and refresher training to HCP (including consultant personnel) about the following: sick leave policies and the importance of not reporting to or remaining at work when sick
Caregivers are trained in the procedure to be taken when a new resident enters the facility (from hospital/household)	The facility has provided education and refresher training to HCP (including consultant personnel) about the following: new policies for source control while in the facility
Facility staff are trained on the procedure to be adopted in case of a new confirmed/suspected COVID-19 host	The facility has provided staff with education on how to use a facemask or respirator if more than source control is required
Specific training was carried out	All HCP are reminded to practice social distancing when in break rooms and common areas
More than 50% of the facility staff has attended the specific training activities	If HCP are ill, they are instructed to keep their cloth face covering or facemask on and leave the facility. HCP with suspected or confirmed COVID-19 should notify their supervisor at any facility where they work

**Table 3:** Characteristics of SARS-CoV-2 cases among facilities with outbreaks during the first pandemic phase.

Variables	Residents		Healthcare Workers		Total	
Age (Mean, SD)	81.0 (±11.0)		47.0 (±10.0)		69.4 (±19.5)	
Gender	*n*	%	*n*	%	*n*	%
Female	101	61.6	66	75.9	167	66.5
Male	63	38.4	21	24.1	84	43.5
**Lethality by group**						
Female	19/101	18.8	0/66	0	19/167	11.4
Male	17/63	27.0	0/21	0	17/84	20.2
>90 years	9/26	34.6	0	0	9/26	34.6
80 to 89 years	21/80	26.3	0	0	21/80	26.3
70 to 79 years	4/28	14.3	0	0	4/28	14.3
60 to 69 years	2/12	16.7	0/10	0	2/22	9.1
<60 years	0/18	0	0/77	0	0/95	0
Total	36/164	22.0	0	0	36/251	14.3
Comorbidities						
No	5	3.1	77	88.5	82	32.7
Unknown	91	55.4	3	3.4	94	37.4
Yes	68	41.5	7	8.1	75	29.9
Distribution of comorbidities						
Cardiovascular diseases	34	50.0	0	0	34	45.3
Chronic Respiratory diseases	18	26.5	3	42.9	21	28.0
Metabolic Diseases	12	17.7	0	0	12	16.0
Kidney diseases	10	14.7	0	0	10	13.3
Cancer	8	11.8	0	0	8	10.7
Diabetes Mellitus	7	10.3	0	0	7	9.3
Chronic neurological diseases	5	7.4	0	0	5	6.7
Obesity (BMI 30 to 40)	3	4.4	0	0	3	4.0
HIV	2	2.9	0	0	2	2.7
Other Conditions	51	75.0	5	71.4	56	74.7

**Table 4:** Results of the introductory questionnaire (*N* = 14).

General Structure	*n*	(%)
Accomodation facility		
Nursing home	2	(14.3%)
Retirement home	12	(85.7%)
Information on the facility, residents and staff	Median	(Range)
Total number of beds	40	(15–163)
Total number of residential rooms	24.5	(12–117)
Total number of single residential rooms	9	(0–64)
Current number of resident guests	35.5	(10–104)
Number of beds predisposed for COVID-19 isolation	2	(0–9)
Number of COVID-19 cases are currently confirmed among residents	0	(0–0)
Number of beds set up for quarantine period and contact monitoring	1	(0–13)
Number of subjects who provide activities in the structure (employees and non-employees.)	17	(11–160)
Number of Nurses in the structure	2	(0–14)
Number of social and health workers employed in the structure	10	(1–70)
Number of COVID-19 cases currently confirmed among facility staff	0	(0–0)
Procedures performed by facilities	*n*	(%)
Fourteen-day observation period for new residents	12	(85.7%)
Nasopharyngeal swab performed at the beginning and at the end of the observation period	12	(85.7%)
Facility staff advised not to attend work if symptomatic	14	(100%)
Body temperature assessment before entering the facility	14	(100%)
All entrances of the facilities are provided with ABHR	14	(100%)
Use of surgical face mask for entire duration of stay (visitors)	14	(100%)
Instructions provided to facility staff on limiting contact if not needed for patient care	14	(100%)
Restrictions on visits by family members	14	(100%)
Daily body temperature measurement for all residents in the facility	13	(92.9%)
Staff informed on facility admission procedures for new residents	14	(100%)
Expected rapid isolation procedure of COVID-19 suspected/confirmed cases and reporting to the referring doctor	14	(100%)
Expected procedure for transferring COVID-19 suspected/confirmed cases in the impossibility of isolation	9	(64.3%)
Information provided to facility staff on the procedures to perform in the event of a suspected/confirmed case	14	(100%)
Dedicated space provided within the facilities for cohort isolation of COVID-19 cases	9	(64.3%)
Member of facility staff identified to verify the correct use of PPE by health workers and proper hand hygiene	12	(85.7%)
Alcohol-based hand rub readily available to all facility staff	14	(100%)
Periodic sanitation of facility staff rooms	14	(100%)
Facility has a person in charge for the control of consumption, purchases and stocks of PPE	14	(100%)
The cleaning staff is:		
Part of the facility workforce	12	(85.8%)
External staff	1	(7.1%)
Partly in the workforce and partly external to the facility	1	(7.1%)
Specific COVID-19 training carried out for staff (HCA, Nurses, etc):		
No	4	(28.6%)
Yes, theoretical	5	(35.7%)
Yes, theoretical–practical	5	(35.7%)
Percentage of the staff who completed dedicated COVID-19 training.		
Less than 25%	0	(0%)
25 to 50%	1	(10%)
51 to 75%	1	(10%)
Over 75%	8	(80%)

**Table 5:** Application of checklist procedures in residential facilities for elderly people in the city of Sassari (*N* = 14).

1. Restrictions on visitors and non-essential personnel	*n*	(%)
Limitation of visits	14	(100%)
Exceptions assessed individually	7	(50%)
Visitor screening	12	(86%)
Visitor behavioral restrictions	12	(86%)
Suspension of non-essential services	14	(100%)
Communication of facility’s lockdown to family members	12	(86%)
Alternative methods to the visit	14	(100%)
Presence of information boards at entrances	3	(21%)
2. Education, Monitoring, and Screening of Healthcare Personnel (HCP)	*n*	(%)
Staff education: COVID-19	6	(43%)
Staff education: Sick leave	14	(100%)
Staff education: New infection control policies	10	(71%)
Monitoring: Hand Hygiene Audit	2	(14%)
Monitoring: PPE Selection and Use Audit	2	(14%)
Monitoring: Cleanliness and Disinfection Audit	2	(14%)
Staffing Needs and Shortage Plan	5	(36%)
Universal use of face masks	12	(86%)
Instruction given to facility staff on different types of masks	6	(43%)
Instruction given to facility staff on social distancing	11	(79%)
Staff screening at the beginning of the work shift	11	(79%)
Information on how to behave in case of symptoms while working in the facility	12	(86%)
Staff symptoms log	6	(43%)
3. Education, Monitoring, and Screening, and Cohorting of Residents	*n*	(%)
Information: COVID-19	14	(100%)
Information: informing facility staff of the onset of symptoms	13	(93%)
Information: protective actions to be implemented	14	(100%)
Information: protective actions implemented by the facility	14	(100%)
Daily monitoring of symptoms	10	(71%)
Application of precautions to suspected COVID-19 cases	13	(93%)
Symptomatic resident log	10	(71%)
Interruption of community activities	5	(36%)
Interruption of communal catering	4	(29%)
Additional actions in emergency: movements restriction	4	(29%)
Additional actions in emergency: movements not restricted but precautions to be taken	6	(43%)
Monitoring sick residents three times a day	12	(86%)
Dedicated COVID-19 area	10	(71%)
Dedicated COVID-19 team	2	(14%)
COVID-19 patient management plan	9	(64%)
New admission/readmission management plan	8	(57%)
Use of recommended PPE in COVID-19 areas or facility-wide if high number of cases	12	(86%)
4. Availability of Personal Protective (PPE) Equipment and other Supplies	*n*	(%)
PPE supply assessment	10	(71%)
Involvement of the Prevention Department for PPE shortages	5	(36%)
PPE supply optimisation measures	7	(50%)
PPE near to the patient care areas	0	(0%)
Availability of virucidal disinfectants	14	(100%)
Wipes/bins available for respiratory hygiene in shared spaces	5	(36%)
5. Infection Prevention and Control Practices	*n*	(%)
Hand hygiene: before resident contact, even if gloves will be worn	12	(86%)
Hand hygiene: after contact with the resident	9	(64%)
Hand hygiene: after contact with blood, body fluids, or contaminated surfaces or equipment	10	(71%)
Hand hygiene: before performing an aseptic task	6	(43%)
Hand hygiene: after removing PPE	11	(79%)
Facility favors use of ABHR	4	(29%)
PPE used in suspected/confirmed cases: gloves	14	(100%)
PPE used in suspected/confirmed cases: coveralls	13	(93%)
PPE used in suspected/confirmed cases: FFP2/FFP3	12	(86%)
PPE used for suspected/confirmed cases: eye protection	12	(86%)
Proper PPE removal and subsequent hand hygiene	11	(79%)
Hand hygiene supply available in care areas	7	(50%)
Monitoring: hand hygiene adherence and correct use of PPE	2	(14%)
Disinfection of shared patient care equipment	11	(79%)
Suitability of disinfectants for environmental cleaning	14	(100%)
Knowledge of contact times of disinfectants	4	(29%)
Use of disinfectant according to label instructions	13	(93%)
6. Communication	*n*	(%)
Public Health Communication: single case suspected or confirmed	13	(93%)
Public Health Communication: worsening of respiratory symptoms	14	(100%)
Public Health Communication: multiple cases of respiratory symptoms	12	(86%)
Procedures for informing family members and staff about suspected/confirmed cases	13	(93%)
Procedures for informing about suspected/confirmed cases during intake from external services.	9	(64%)

## Data Availability

The data presented in this study are available on reasonable request from the corresponding author.

## Supplementary Materials

The following are available online at https://www.mdpi.com/article/10.3390/ijerph18115712/s1, Table S1: Introductory questionnaire for the assessment of “Preparedness” in residential facilities for the elderly in the city of Sassari.
