# Supplementary material for: SARS-CoV-2 Epidemics in Retirement and Nursing Homes in Italy: A New Preparedness Assessment Model after the First Epidemic Wave
Source: Int J Environ Res Public Health. 2021 May 26;18(11):5712. doi: 10.3390/ijerph18115712 (PMC8199425; doi:10.3390/ijerph18115712)
Supplement: Supplementary file 1 [file ijerph-18-05712-s001.zip › ijerph-1161370-S1.pdf]

Table S1. Introductory questionnaire for the assessment of "Preparedness" in residential facilities for the elderly in the city of Sassari

General information

Name of the facility:	________________________
Name of the facility manager:	________________________
Telephone number of the facility:	________________________
E-mail address of the facility:	________________________
Date of the survey:	________________________

Information on the facility, residents and workers

Total number of beds:	________________________
Total number of residential rooms:	________________________
Total number of residential single rooms:	________________________
Current number of residents:	________________________
Number of beds provided for isolation of COVID-19 cases according to facility plan:	________________________
Number of confirmed COVID-19 cases currently present among residents:	________________________
Number of beds provided for quarantine (new residents admissions; contacts of suspected/confirmed COVID-19 cases) according to facility plan:	________________________
Number of people working in the facility (employees and non-employees):	________________________
Number of Nurses among the staff in the facility:	________________________
Number of Healthcare assistants among the staff in the facility:	________________________
Number of confirmed COVID-19 cases currently present among facility staff:	________________________

Procedures

Are new residents housed in a dedicated room to avoid contact with other residents during the observation period (14 days)?	|__|Yes	|__|No
Are new residents tested for Covid19 at the time of admission and at the end of the observation period (14 days)?	|__|Yes	|__|No

Is the facility staff informed that they should not come to work with fever, myalgia, profound asthenia, or respiratory/gastrointestinal symptoms?	|__|Yes	|__|No
Is a body temperature screening provided to all staff members prior to access to the facility?	|__|Yes	|__|No
Is Alcohol-based hand rubs (ABHR) available for anyone at the facility entrance?	|__|Yes	|__|No
Does anyone entering the facility wear a surgical mask the whole time of the stay?	|__|Yes	|__|No
Is the facility staff informed to avoid close contact with co-workers, except for any nursing activities that require it out?	|__|Yes	|__|No
Is there a procedure in place regarding limiting visits to residents by family members?	|__|Yes	|__|No
Is daily body temperature measurement provided to all facility residents?	|__|Yes	|__|No
Is nursing staff instructed on the procedure to be followed when a new resident (from hospital/household) is admitted?	|__|Yes	|__|No
Is there a procedure for rapid isolation and notification to the referring physician of a new confirmed/suspected COVID-19 case?	|__|Yes	|__|No
Is there a provision for the transfer of the confirmed/suspected case to another facility if adequate isolation of the subject cannot be arranged?	|__|Yes	|__|No
Is nursing staff informed on the procedure to be taken in the event of a new confirmed/suspected COVID-19 case?	|__|Yes	|__|No
Has a separate facility area been identified to be used if there is a high number of COVID-19 cases (cohort isolation)?	|__|Yes	|__|No
Has a staff member been identified in charge of proper use of PPE and proper hand hygiene by health care workers?	|__|Yes	|__|No
Is ABHR present and readily available to all staff during care activities?	|__|Yes	|__|No
Is periodic sanitization of workers' ancillary rooms performed?	|__|Yes	|__|No
Is there a person in charge of monitoring PPE inventory, consumption, and orders?	|__|Yes	|__|No

The cleaning staff is:	|__|	Internal facility staff
	|__|	External facility staff
	|__|	Both internal and external staff
Have any members among healthcare personnel (nurses, healthcare assistants, physicians) currently received specific training related to COVID-19, in accordance with the facility's recommendations?	|__|	No
	|__|	Yes, theoretical training (dedicated online courses, dedicated lessons)
	|__|	Yes, both theoretical and practical training (including simulations of PPE wearing and proper hand hygiene)
Percentage of the facility staff who have taken part in the training (physicians, nurses, healthcare assistants). [Supplementary question, depending on the answer to the previous question].	|__|	< 25%
	|__|	25-50%
	|__|	51-75%
	|__|	>75%
